# Mu opioid receptor: from pain to glucose metabolism

**DOI:** 10.18632/oncotarget.14231

**Published:** 2016-12-26

**Authors:** Eva Tudurí, Ruben Nogueiras

**Affiliations:** ^1^ Instituto de Investigaciones Sanitarias (IDIS), CIMUS, University of Santiago de Compostela, Santiago de Compostela, Spain; CIBER Fisiopatología de la Obesidad y Nutrición (CIBERobn), Santiago de Compostela, Spain; ^2^ Instituto de Investigaciones Sanitarias (IDIS), CIMUS, University of Santiago de Compostela, Santiago de Compostela, Spain; CIBER Fisiopatología de la Obesidad y Nutrición (CIBERobn), Santiago de Compostela, Spain

**Keywords:** mu opioid receptor, brain, glucose, insulin, sympathetic nervous system

The prevalence of type 2 Diabetes Mellitus (T2DM) is increasing worldwide and it is mainly due to the rise in obesity caused by modern life [1]. The foremost feature of T2DM is uncontrolled blood glucose levels, and recurrent hyperglycemia episodes may lead to severe healthy complications. Thus, a better understanding of the mechanisms that govern glucose metabolism is necessary to design therapeutic strategies to maintain blood glucose within a physiological range. Those mechanisms may act specifically on key peripheral tissues involved in the control of systemic glucose homeostasis such as the islets of Langerhans (responsible for producing and secreting insulin and glucagon, among other hormones), liver, adipose tissue or skeletal muscle. Yet other mechanisms of regulation of postprandial glucose act through the central nervous system (CNS). Following food ingestion, the CNS receives input about the body’s nutritional status and subsequently orchestrates changes in peripheral tissues to maintain glucose homeostasis. As a matter of fact, brain neurons respond to multiple hormones, glucose and fatty acids controlling glucose metabolism in a food intake- and body weight-independent fashion [2].

The opioid system, constituted by the opioid receptors delta (δ-, DOR), kappa (κ-, KOR) and mu (μ-, MOR) and their endogenous ligands (dynorphins, enkephalins and endorphins respectively), is widely distributed throughout the CNS. Several reports have investigated the role of MOR in insulin sensitivity; but, the results are somehow controversial. Mice lacking MOR in the whole body exhibited enhanced glucose tolerance that results from insulin hypersecretion [3, 4]. However, pharmacological studies performed in insulin resistant animal models demonstrated improved insulin sensitivity upon peripheral MOR activation [5]. At the central level, the opioid system is well-known for its pain-relieving and addictive properties as well as for the control of food intake, and, importantly, convincing results have emerged in the last years demonstrating its participation in other biological processes such as energy expenditure and lipid metabolism [6].

**Figure:**
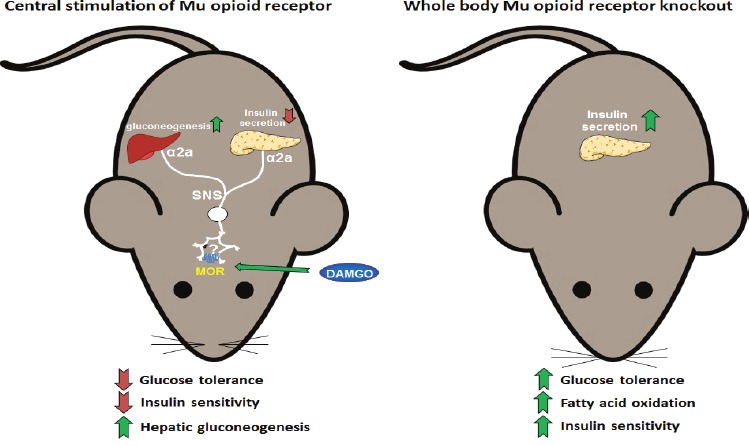
Schematic representation of the actions of mu opioid receptor on insulin and glucose The central stimulation of the mu opioid receptor (left panel) impairs glucose tolerance and insulin sensitivity and stimulates hepatic gluconeogenesis. These effects are mediated via the alpha-2a adrenergic receptor of the sympathetic nervous system. On the other hand, mice lacking of mu opioid receptor in the whole body (right panel) showed enhanced glucose tolerance and insulin sensitivity as well as increased fatty acid oxidation when fed a high fat diet.

Conducting a series of *in vivo* experiments by means of pharmacological approaches and genetically modified animals, we have recently unraveled the role of central opioid receptors in the regulation of glucose homeostasis. While a single intracerebroventricular (i.c.v.) injection of specific agonists for KOR and DOR did not produce any effect on glucose tolerance, the i.c.v. injection of the MOR agonist DAMGO dose-dependently impaired the glucose excursions [7]. This is likely the result of several events observed that include blunted glucose-stimulated insulin secretion (GSIS), decreased insulin sensitivity and up-regulated liver expression of the gluconeogenic genes *Pck-1* and *G6pc* [7]. Exploring the mechanisms underlying these outcomes, we found that the activation of central MOR failed to impair GSIS and glucose tolerance in mice with pharmacologically blocked α_2A-_ adrenoceptors as well as in α_2A-_ adrenoceptor knockout mice [7]; hence, the effects of central MOR signaling on insulin-secreting β-cells are mediated via sympathetic innervation.

Although we have conducted pharmacological approaches, our results indicating that central DAMGO induces glucose intolerance support previous findings where mice lacking MOR showed enhanced glucose tolerance [3, 4]. Therefore, this suggests that even though MOR is expressed in the islets of Langerhans and directly controls insulin secretion from pancreatic β-cells [3], brain MOR might have, at least partially, a physiological role in the control of glucose homeostasis. Since previous pharmacological studies activating MOR at peripheral level improved insulin sensitivity in rodents [5] there is also the possibility that peripheral and brain MOR exert opposite effects on glucose metabolism.

Furthermore, given the complexity and plasticity of the numerous neuronal pathways controlling glucose metabolism, it will be also important to identify the neuronal populations by which central MOR regulate hormone secretion from islets and insulin sensitivity in peripheral tissues. In this sense, it is well established that the hypothalamus is a key center for the regulation of glucose metabolism, and MOR is located in several hypothalamic areas [8]. It is therefore tempting to speculate that some of these hypothalamic neurons expressing MOR may be responsible for the maintenance of glucose homeostasis. In particular, since the hypothalamus regulates the activity of sympathetic outflow neurons to pancreas.

Having demonstrated that central MOR causes glucose intolerance, the key question is the validity of this route to improve glucose tolerance. Unfortunately, our first results using i.c.v. β-FNA, a MOR specific and irreversible antagonist, showed no effects in two different mouse models displaying glucose intolerance [7]. One explanation for this might be the reduced hypothalamic MOR levels in obese and insulin resistant mice, making the subsequent inhibition of MOR likely insufficient to ameliorate glucose intolerance in these mice. Further studies investigating other MOR antagonists or the genetic inhibition of this receptor in specific brain areas of glucose intolerant mice may help to clarify this aspect.

In summary, the intriguing role of brain MOR on glucose metabolism expands our knowledge on the “classical” biological actions regulated by the opioid system, whose complexity and functionality are still beyond our understanding.
